# Supplementary material for: Pyruvate Dehydrogenase Kinase 1 inhibition mediated oxidative phosphorylation enhancement in cartilage promotes osteoarthritis progression
Source: BMC Musculoskelet Disord. 2023 Jul 20;24:597. doi: 10.1186/s12891-023-06585-6 (PMC10357736; doi:10.1186/s12891-023-06585-6)
Supplement: Supplementary file 1 — Additional file 1. [file 12891_2023_6585_MOESM1_ESM.zip › KEGG.Gsea.1653622432464/KEGG_PROTEIN_EXPORT.html]

Details for gene set KEGG\_PROTEIN\_EXPORT[GSEA]

|  || Dataset | 55457\_collapsed\_to\_symbols.cls.cls #OA\_versus\_normal.cls.cls #OA\_versus\_normal\_repos |
| Phenotype | cls.cls#OA\_versus\_normal\_repos |
| Upregulated in class | OA |
| GeneSet | KEGG\_PROTEIN\_EXPORT |
| Enrichment Score (ES) | 0.26675576 |
| Normalized Enrichment Score (NES) | 0.8481212 |
| Nominal p-value | 0.6516854 |
| FDR q-value | 0.9188281 |
| FWER p-Value | 1.0 |
Table: GSEA Results Summary

  

Fig 1: Enrichment plot: KEGG\_PROTEIN\_EXPORT      
 Profile of the Running ES Score & Positions of GeneSet Members on the Rank Ordered List

  

| SYMBOL | TITLE | RANK IN GENE LIST | RANK METRIC SCORE | RUNNING ES | CORE ENRICHMENT || 1 | SPCS1 | signal peptidase complex subunit 1 [Source:HGNC Symbol;Acc:HGNC:23401] | 303 | 0.747 | 0.1375 | Yes |
| 2 | SEC61G | SEC61 translocon subunit gamma [Source:HGNC Symbol;Acc:HGNC:18277] | 1163 | 0.485 | 0.1752 | Yes |
| 3 | SEC62 | "SEC62 homolog, preprotein translocation factor [Source:HGNC Symbol;Acc:HGNC:11846]" | 1272 | 0.464 | 0.2668 | Yes |
| 4 | SPCS3 | signal peptidase complex subunit 3 [Source:HGNC Symbol;Acc:HGNC:26212] | 2772 | 0.273 | 0.2092 | No |
| 5 | SEC11A | "SEC11 homolog A, signal peptidase complex subunit [Source:HGNC Symbol;Acc:HGNC:17718]" | 5314 | 0.046 | 0.0219 | No |
| 6 | SEC61A1 | SEC61 translocon subunit alpha 1 [Source:HGNC Symbol;Acc:HGNC:18276] | 5378 | 0.041 | 0.0260 | No |
| 7 | SRP9 | signal recognition particle 9 [Source:HGNC Symbol;Acc:HGNC:11304] | 5498 | 0.033 | 0.0238 | No |
| 8 | OXA1L | OXA1L mitochondrial inner membrane protein [Source:HGNC Symbol;Acc:HGNC:8526] | 5974 | -0.003 | -0.0125 | No |
| 9 | SRPRA | SRP receptor subunit alpha [Source:HGNC Symbol;Acc:HGNC:11307] | 6566 | -0.047 | -0.0482 | No |
| 10 | SRP19 | signal recognition particle 19 [Source:HGNC Symbol;Acc:HGNC:11300] | 6945 | -0.075 | -0.0613 | No |
| 11 | SPCS2 | signal peptidase complex subunit 2 [Source:HGNC Symbol;Acc:HGNC:28962] | 6981 | -0.078 | -0.0472 | No |
| 12 | SRPRB | SRP receptor subunit beta [Source:HGNC Symbol;Acc:HGNC:24085] | 7553 | -0.123 | -0.0649 | No |
| 13 | SEC63 | "SEC63 homolog, protein translocation regulator [Source:HGNC Symbol;Acc:HGNC:21082]" | 8151 | -0.167 | -0.0753 | No |
| 14 | SRP72 | signal recognition particle 72 [Source:HGNC Symbol;Acc:HGNC:11303] | 8338 | -0.181 | -0.0507 | No |
| 15 | SEC61B | SEC61 translocon subunit beta [Source:HGNC Symbol;Acc:HGNC:16993] | 8510 | -0.194 | -0.0221 | No |
| 16 | SRP14 | signal recognition particle 14 [Source:HGNC Symbol;Acc:HGNC:11299] | 9288 | -0.255 | -0.0275 | No |
| 17 | HSPA5 | heat shock protein family A (Hsp70) member 5 [Source:HGNC Symbol;Acc:HGNC:5238] | 10820 | -0.407 | -0.0585 | No |
| 18 | SEC61A2 | SEC61 translocon subunit alpha 2 [Source:HGNC Symbol;Acc:HGNC:17702] | 11352 | -0.479 | 0.0034 | No |
| 19 | SRP54 | signal recognition particle 54 [Source:HGNC Symbol;Acc:HGNC:11301] | 11754 | -0.541 | 0.0890 | No |
Table: GSEA details [plain text format]

  

Fig 2: KEGG\_PROTEIN\_EXPORT      
 Blue-Pink O' Gram in the Space of the Analyzed GeneSet

  

Fig 3: KEGG\_PROTEIN\_EXPORT: Random ES distribution      
 Gene set null distribution of ES for **KEGG\_PROTEIN\_EXPORT**
